# Molecular epidemiology and characterization of antimicrobial-resistant *Staphylococcus haemolyticus* strains isolated from dairy cattle milk in Northwest, China

**DOI:** 10.3389/fcimb.2023.1183390

**Published:** 2023-05-17

**Authors:** Muhammad Shoaib, Jie Xu, Xiaoqin Meng, Zhongyong Wu, Xiao Hou, Zhuolin He, Ruofeng Shang, Hongjuan Zhang, Wanxia Pu

**Affiliations:** ^1^ Key Laboratory of New Animal Drug Project, Gansu Province/Key Laboratory of Veterinary Pharmaceutical Development, Ministry of Agriculture and Rural Affairs, Lanzhou Institute of Husbandry and Pharmaceutical Sciences of Chinese Academy of Agricultural Sciences (CAAS), Lanzhou, China; ^2^ Lanzhou Center for Animal Disease Control and Prevention, Lanzhou, China

**Keywords:** dairy mastitis, *Staphylococcus haemolytius*, antimicrobial resistance, phylogeny, sequence typing, multi-drug resistance (MDR)

## Abstract

**Introduction:**

Non-aureus *Staphylococcus* (NAS) species are currently the most commonly identified microbial agents causing sub-clinical infections of the udder and are also deemed as opportunistic pathogens of clinical mastitis in dairy cattle. More than 10 NAS species have been identified and studied but little is known about *S. haemolyticus* in accordance with dairy mastitis. The present study focused on the molecular epidemiology and genotypic characterization of *S. haemolyticus* isolated from dairy cattle milk in Northwest, China.

**Methods:**

In this study, a total of 356 milk samples were collected from large dairy farms in three provinces in Northwest, China. The bacterial isolation and presumptive identification were done by microbiological and biochemical methods following the molecular confirmation by 16S rRNA gene sequencing. The antimicrobial susceptibility testing (AST) was done by Kirby-Bauer disk diffusion assay and antibiotic-resistance genes (ARGs) were identified by PCR. The phylogenetic grouping and sequence typing was done by Pulsed Field Gel Electrophoresis (PFGE) and Multi-Locus Sequence Typing (MLST) respectively.

**Results:**

In total, 39/356 (11.0%) were identified as positive for *S. haemolyticus*. The overall prevalence of other *Staphylococcus* species was noted to be 39.6% (141/356), while the species distribution was as follows: *S. aureus* 14.9%, *S. sciuri *10.4%, *S. saprophyticus* 7.6%, *S. chromogenes* 4.2%, *S. simulans* 1.4%, and *S. epidermidis* 1.1%. The antimicrobial susceptibility of 39 *S. haemolyticus* strains exhibited higher resistance to erythromycin (92.3%) followed by trimethoprim-sulfamethoxazole (51.3%), ciprofloxacin (43.6%), florfenicol (30.8%), cefoxitin (28.2%), and gentamicin (23.1%). All of the *S. haemolyticus* strains were susceptible to tetracycline, vancomycin, and linezolid. The overall percentage of multi-drug resistant (MDR) *S. haemolyticus* strains was noted to be 46.15% (18/39). Among ARGs, *mphC* was identified as predominant (82.05%), followed by *ermB* (33.33%), *floR* (30.77%), *gyrA* (30.77%), *sul1* (28.21%), *ermA* (23.08%), *aadD* (12.82%), *grlA* (12.82%), *aacA-aph*D (10.26%), *sul2* (10.26%), *dfr*A (7.69%), and *dfr*G (5.13%). The PFGE categorized 39 *S. haemolyticus* strains into A-H phylogenetic groups while the MLST categorized strains into eight STs with ST8 being the most predominant while other STs identified were ST3, ST11, ST22, ST32, ST19, ST16, and ST7.

**Conclusion:**

These findings provided new insights into our understanding of the epidemiology and genetic characteristics of *S. haemolyticus* in dairy farms to inform interventions limiting the spread of AMR in dairy production.

## 1 Introduction

Bovine mastitis is the most common and economically deadly disease impacting the dairy sector all over the world (Gussmann et al., 2019). The incidence of clinical mastitis (CM) on large dairy farms in China could reach up to 3.3% per month (Dallago et al., 2021). Clinical mastitis is caused by numerous pathogens, but *Staphylococcus* species classified as coagulase-positive *Staphylococcus* including *S. aureus* and coagulase-negative *Staphylococcus* (CNS), also known as non-aureus *Staphylococcus* (NAS), are the most common pathogens in lactating dairy cows (De Buck et al., 2021). NAS is one of the most important and widespread groups of pathogens including numerous species such as *S. chromogenes, S. haemolyticus, S. sciuri, S. saprophyticus, S. simulans, S. succinus, and S. epidermidis* (Wald et al., 2019). NAS pathogens are not only opportunistic in animals and humans but are also widely distributed in the environment such as dust, soil, water, and air (Wu et al., 2021). Although NAS species are considered to be a pathogen of low clinical incidence, it has recently been identified as the relatively common causative agent of bovine mastitis in many countries including China (Al-Harbi et al., 2021). Intra-mammary infection (IMI) with NAS species is often associated with asymptomatic mastitis leading to an increase in somatic cell count and sometimes severe mastitis resulting in reduced milk yield (Adkins et al., 2018). Among NAS, *S. haemolyticus* ranked second in terms of bacterial recovery and is one of the important opportunistic pathogenic bacteria (Jenkins et al., 2019).

Mastitis remains one of the most common reasons for antimicrobial, especially antibiotics, use in dairy herds worldwide (Hogeveen et al., 2011). The higher prevalence of multidrug-resistant strains in livestock has led to the extensive use of antimicrobials to treat multiple diseases on dairy farms in China (Tang et al., 2017). *Staphylococcus* species including NAS can develop resistance to multiple antimicrobials, thus reducing the effectiveness of antimicrobial therapy (Jenkins et al., 2019). The most common antimicrobial resistance genes (ARGs) detected among different classes of antimicrobials include β-lactam-resistance genes (*blaZ* and *mecA*), tetracycline-resistance genes (*tet*O, *tet*K, *tet*M, and *tet*L), aminoglycoside-resistance genes (*aad*D, *aph*A3, and *aac*A*-aph*D), and macrolide-lincosamide resistance genes (*erm*A*, erm*B*, erm*C*, erm*T*, msr*A*, mph*C, and *lnu*A) (Pizauro et al., 2019; Qu et al., 2019; Pérez et al., 2020; Walid et al., 2021). Multidrug-resistant NAS strains are increasingly reported, and the growing resistance of NAS agents to antimicrobials also limits the choice of drugs for treatment purposes (Schoenfelder et al., 2017). NAS species are also recognized as reservoirs of drug-resistance genes, highlighting their threat to public health (Osman et al., 2017). Molecular epidemiological studies by multiplex PCR, pulse field gel electrophoresis (PFGE), 16S rRNA gene sequencing, and multi-locus sequence typing (MLST) are important in understanding the source and route of transmission of these pathogens (Kim et al., 2019; Lin et al., 2022).

Because of the ubiquitous nature and acquisition of ARGs by these microbial agents, it is important to understand their epidemiology through the molecular approaches mentioned above (Srednik et al., 2017; Benites et al., 2021). The Northwest region of China is an important agricultural and pastoral region with much potential for the dairy industry in Gansu, Ningxia, Qinghai, and Xinjiang provinces, and the status of AMR among NAS, particularly *S. haemolyticus* isolated from the dairy herd is still unknown in the northwest region of China. Therefore, the present study was designed to determine the molecular epidemiology and antimicrobial resistance traits of *S. haemolyticus* isolated from dairy origin in Northwest, China. This study also focused on the genotypic characterization of *S. haemolyticus* strains concerning phylogenetic lineage and sequence types (STs).

## 2 Materials and methods

### 2.1 Materials

Antibiotic disks used for AST were purchased from Shanghai Kejia Drug Testing Equipment Co., Ltd; *Sma*I, *Xba*I, proteinase K, and La Taq DNA polymerase from Takara Co., Ltd; Lysozyme and lysostaphin from Sigma Aldrich, Beijing, China; Mueller-Hinton (MH) and Mannitol Salt agar from Guangdong Huankai Microbial Technology Co., Ltd; Agarose from Shanghai Shenggong Biological Co., Ltd; SeaKem Gold Agarose from Lonza Co., Ltd; and bacterial genomic DNA extraction kit from Tiangen Biochemical Technology Co., Ltd. The quality control strain used in this experiment was *Staphylococcus haemolyticus* ATCC25923, obtained from a culture bank.

### 2.2 Sample collection and transportation

A total of n = 356 milk samples were collected from five large dairy farms in five cities of three provinces [Gansu (Lanzhou, LZ n= 96; Zhangye, ZY n = 57), Qinghai (Xining, XN n = 48; Yushu, YS n = 71), Ningxia, NX n = 84] in the Northwest region of China. The milk samples were drawn directly from the animal teats into sterile 50 mL tubes. Before taking the sample, the animal udder was washed with lukewarm water and dried with a clean towel. The teats of animals were swabbed with 0.5% iodine solution (Merck, Germany), and the hands of the milking personnel were also washed with an antiseptic solution. The first few streaks of milk were discarded, and the middle streaks were collected as adopted by Aqib et al., 2021. The collected milk samples were stored at 4°C and transported to the microbiology laboratory for further processing.

### 2.3 Isolation and identification of *Staphylococcus* species

In this study, 100 μL of collected milk samples were cultured on blood agar supplemented with 5% sheep blood. The agar plates were incubated at 37°C for 24 hours and hemolysis patterns were observed. Further, isolates were purified, and differentiated by culturing on mannitol salt agar following the prior incubation conditions, and presumptive identification was done by microbiological examination based on colony morphology, gram staining, catalase, and coagulase tests as done previously (Gizaw et al., 2020). At the same time, all isolates were identified by the automatic biochemical identification VITEK-2 system (BioMérieux, France) according to the prior recommendations (Chajęcka-Wierzchowska et al., 2023). Furthermore, the molecular confirmation of the target specie, *S. haemolyticus* strains was done by 16S rRNA gene sequencing according to the previously described protocol (Qu et al., 2019). Briefly, the genomic DNA was extracted using genomic DNA Extraction Kit (Tiangen Biochemical Technology Co., Ltd) and PCR was performed using the primers mentioned in **Table S1**. The PCR reaction mixture (25 μL) comprised 12.5 μL Master Mix, 1 μL of forward and reverse primer each, 1 μL of extracted DNA, and 9.5 μL of deionized water. The PCR amplification conditions were adjusted as initial denaturation at 95°C for 5 minutes, followed by 35 cycles of complete denaturation at 95°C for 30 seconds, elongation at 55°C for 30 seconds, and extension at 74°C for 1 minute with a final extension at 74°C for 5 minutes. The positive (*S. haemolyticus* ATCC25923) and negative (without genomic DNA) control PCR were done to validate the results. The PCR product was visualized on 1% agarose gel under the GelDoc XR system and purified with Wizard^®^ genomic DNA purification kit (Promega, USA) following the guidelines of the manufacturer and then sent elsewhere (Beijing Huada Gene Technology Co., Ltd) for sequencing analysis. The sequencing data were analyzed by checking the sequence similarity index ≥99.0% and submitted to the National Center for Biotechnology Information (NCBI) GenBank (https://blast.ncbi.nlm.nih.gov/) under the accession numbers from OQ652547 to OQ652584 and OQ787674.

### 2.4 Antimicrobial susceptibility testing of *S. haemolyticus* strains

The antimicrobial susceptibility of the 16S rRNA gene confirming *S. haemolyticus* strains was assessed on the Mueller Hinton agar (MHA) by Kirby-Bauer disk diffusion (KBDD) assay following the EUCAST guidelines against nine antimicrobial agents belonging to nine different classes. The tested antibiotics included were cefoxitin (CFX, 30μg), ciprofloxacin (CIP, 5μg), gentamicin (GEN, 10μg), tetracycline (TET, 30μg), trimethoprim-sulfamethoxazole (SXT, 1.25-23.75μg), florfenicol (FFC, 30μg), erythromycin (ERY, 15μg), and linezolid (LZD, 10μg). The AST of vancomycin was done by MIC broth micro-dilution assay according to the protocol described in the EUCAST guidelines (EUCAST, 2019). Briefly, inoculum for KBDD was prepared by adjusting the turbidity at 0.5 McFarland Standard and swabbing was done on MHA under sterile conditions. Antibiotic disks were placed on agar surface with sterile forcep at the proper distance, and plates were incubated at 35 ± 1°C for 18 ± 2h. After incubation, the zone of inhibition was measured and compared with EUCAST clinical breakpoints (EUCAST, 2019).

### 2.5 Identification of ARGs

All of the 39 strains were screened for the detection of ARGs by PCR. A total of 30 ARGs against nine antibiotics belonged to nine different antimicrobial classes; *erm*A, *erm*B, *erm*C, *erm*F, *erm*(33), *mph*C, and *msr*B for erythromycin (macrolides, lincosamides, and streptogramin B); *van*A and *van*B for vancomycin (glycopeptide); *cfr* for linezolid (oxazolidinones); *cfx*A for cefoxitin (cephalosporin); *tet*M, *tet*O, *tet*L, and *tet*K for tetracycline (tetracycline’s); *fex*A and *flo*R for florfenicol (amphenicol); *aac*A-*aph*D and *aad*D for gentamicin (aminoglycosides); *gyr*A, *gyr*B, *grl*A, and *grl*B for ciprofloxacin (fluoroquinolones); and *sul1*, *sul2*, *sul3*, *dfr*A, *dfr*D, *dfr*G, and *dfr*K for trimethoprim-sulfamethoxazole (sulfonamides) were screened by PCR amplification using the primer mentioned previously (**Table S2**). The genomic DNA was extracted using the DNA extraction kit and PCR reaction mixture (25 µL) comprising 12.5 µL Master Mix, 1 µL of each primer, 1 µL genomic DNA, and 9.5 µL of deionized water. The PCR amplification conditions were adjusted as 95°C for 5 min for initial denaturation, followed by 30 cycles at 95°C for 30 s, 30 cycles of annealing at various temperatures for 30 s (**Table S2**), initial extension at 72°C for 1 min, and followed by final elongation at 72°C for 10 min. The PCR product was run on 1% agarose gel at 80V and 130mA for 1 hour for visualization under UV light by ethidium bromide staining.

### 2.6 Pulsed-field gel electrophoresis

All 39 *S. haemolytic*us strains were also subjected to PFGE analysis to determine their phylogenetic grouping as described previously (Kim et al., 2019). Briefly, the bacterial culture grown overnight was embedded with low-melting SKG agarose gel, and then blocks were placed in 1×TE buffer containing 10 mg/L lysostaphin and incubated at 37°C for 3.5h. Subsequently, the blocks were digested with *Sma*I at 30°C for 3h. Then, the blocks were loaded into 1% SKG gel, and gel electrophoresis was performed in 0.5×TBE buffer for 18h with a 120° change angle, 6 V/cm voltage, and switching time of 5-40 sec. *Salmonella* H9812 (*Xba*I digestion) was used as size markers. After electrophoresis, the gel was stained with ethidium bromide (1: 10000) for 30 min, decolored in deionized water for 30 min, and photographed under a gel imaging system. The phylogenetic analysis of the PFGE band patterns was done by PyElph software and compared with the standard (Pavel and Vasile, 2012).

### 2.7 Multi-locus sequence typing

MLST analysis was done based on primer sequences published on the MLST website (https://pubmlst.org/shaemolytic/) for seven housekeeping genes of *S. haemolyticus* including *arc haemo*, *SH*1200, *hem*H, *leu*B, *SH*1431, *cfx*E, and *ribose*ABC. The seven housekeeping genes were PCR amplified and sequenced using the genomic DNA of *S. haemolyticus* as a template. The sequencing data were submitted to the MLST database (http://www.pubmlst.net/databases/) to determine the STs of *S. haemolyticus*. The alignment of obtained sequences was done using ChromasPro computer software and the phylogenetic tree was made by the Neighbor-Joining Method in the MEGA-X software.

## 3 Results

### 3.1 Prevalence of *S. haemolyticus* and other *Staphylococcus* species

**Figure 1:**
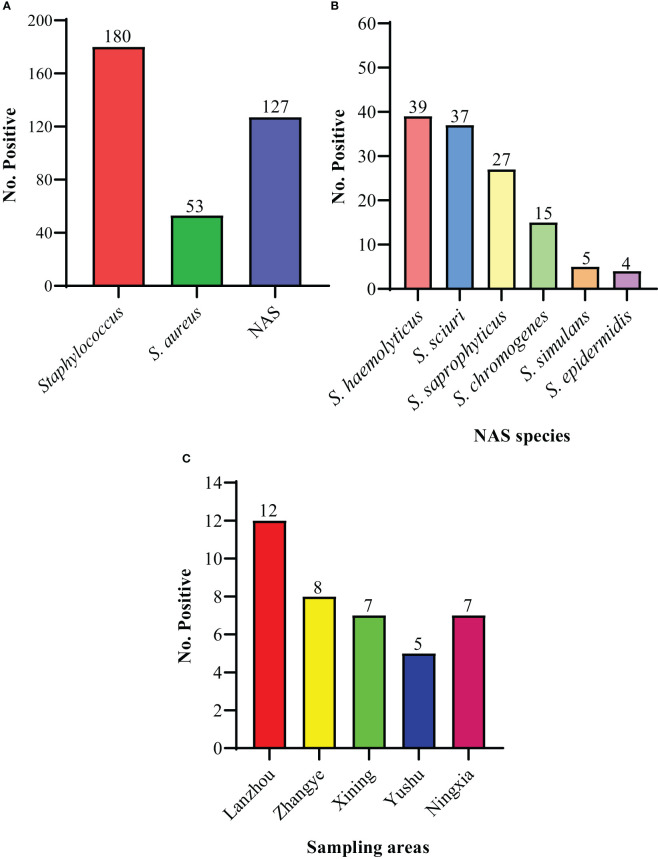
The prevalence of isolated species. **(A)** The overall prevalence of *Staphylococcus*, *S. aureus*, and non-aureus *Staphylococcus* (NAS) species. **(B)** The individual distribution of NAS species. **(C)** The distribution of 39 *S. haemolyticus* strains within sampling cities.

For the current study, a total of 356 milk samples were collected from different dairy farms in different cities (Lanzhou, Zhangye, Xining, Yushu, and Ningxia) in Northwest, China. In the present study, 180/356 (50.6%) isolates were recovered and identified as *Staphylococcus* species. Moreover, the prevalence of *S. aureus* was noted to be 14.9% (53/356) and NAS was 35.7% (127/356) (**Figure 1A**). Among NAS, *S. haemolyticus* was identified as the predominant specie 11.0% (39/356), followed by *S. sciuri* 10.4% (37/356), *S. saprophyticus* 7.6% (27/356), *S. chromogenes* 4.2% (15/356), *S. simulans* 1.4% (5/356), and *S. epidermidis* 1.1% (4/356) (**Figure 1B**). The prevalence of *S. haemolyticus* was higher (14.6%; 7/48) in Xining, followed by Zhangye (14.0%; 8/57), Lanzhou (12.5%; 12/96), Ningxia (8.3%; 7/84), and was lower in Yushu 7.04% (5/71) (**Figure 1C**). The overall prevalence of all other *Staphylococcus* species was noted to be higher in Lanzhou (58.3%; 56/96), followed by Ningxia 48.8% (41/84), Xining 29.2% (14/48), Yushu 28.2% (20/71), and Zhangye 17.5% (10/57). Moreover, the prevalence of *S. aureus* was noted to be 26.0%, 10.5%, and 14.1% in Lanzhou, Zhangye, and Yushu, respectively, while *S. saprophyticus* was higher in Xining (12.5%) and Ningxia (15.5%). The prevalence of other *Staphylococcus* species among different sampling cities is presented in **Table 1**.

**Table 1:** The distribution of other *Staphylococcus* species in different sampling cities.

Species	LZ (n= 96) No. (%)	ZY (n= 57) ^*^No. (%)	YS (n= 71) ^*^No. (%)	XN (n= 48) ^*^No. (%)	NX (n= 84) ^*^No. (%)
*S. aureus*	25 (26.0)	6 (10.5)	10 (14.1)	3 (6.25)	9 (10.7)
*S. sciuri*	15 (15.6)	3 (5.3)	6 (8.4)	2 (4.2)	11 (13.1)
*S. saprophyticus*	6 (6.20)	0 (0.0)	2 (2.8)	6 (12.5)	13 (15.5)
*S. chromogenes*	7 (7.29)	0 (0.0)	0 (0.0)	2 (4.2)	6 (7.1)
*S. simulans*	2 (2.08)	1 (1.75)	0 (0.0)	1 (2.1)	1 (1.2)
*S. epidermidis*	1 (1.04)	0 (0.0)	2 (2.8)	0 (0.0)	1 (1.2)
Total	56 (58.3)	10 (17.5)	20 (28.2)	14 (29.2)	41 (48.8)

LZ, Lanzhou; ZY, Zhangye; YS, Yushu; XN, Xining; and NX, Ningxia.

### 3.2 Phenotypic AMR characteristics of 39 *S. haemolyticus* strains

**Figure 2:**
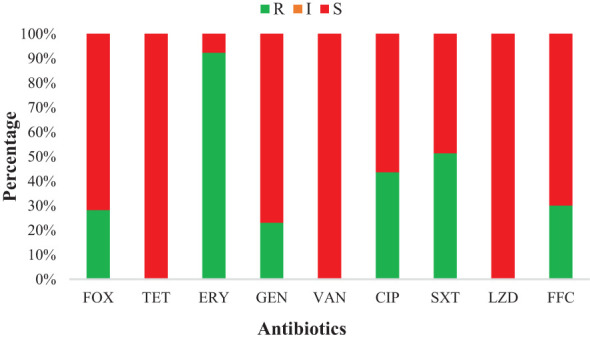
Antimicrobial susceptibility of various antibiotics against *S. haemolyticus* strains of dairy origin: FOX, Cefoxitin; ERY, Erythromycin; TET, Tetracycline; GEN, Gentamicin; CIP, Ciprofloxacin; VAN, Vancomycin; SXT, Trimethoprim-Sulfamethoxazole; FFC, Florfenicol; and LZD, Linezolid.

Most of the *S. haemolyticus* strains showed resistance to ERY (92.3%, 36/39), followed by SXT (51.3%, 20/39), CIP (43.6%, 17/39), FFC (30.8%, 12/39), FOX (28.2%, 11/39), and GEN (23.1%, 9/39). However, all strains were susceptible to TET, VAN, and LZD. Moreover, none of the strains showed intermediate susceptibility to all antibiotics (**Figure 2**). *S. haemolyticus* strains from LZ, YS, and NX showed 100% resistance to ERY, which was noted to be lower in other cities (85.7%, ZY, and 75.0%, XN). The AMR against SXT was noted at 50%, 42.8%, 87.5%, 20.0%, and 42.8% by *S. haemolyticus* strains from LZ, ZY, XN, YS, and NX, respectively (**Table 2**). Moreover, none of the strains from ZY and NX showed resistance to FOX with a similar trend from XN and NX against GEN. It was observed that 80% of the strains from Yushu City were resistant to multiple antimicrobials (FOX+GEN+CIP+FFC) and 20% to SXT. All of the strains from all sampling cities were susceptible to TET, VAN, and LZD. The overall AMR was noted to be higher in strains from YS, followed by NX, LZ, XN, and ZY (**Table 2**).

**Table 2:** The percentage distribution of antimicrobial-resistant and susceptible strains among different sampling cities.

Antibiotics	Lanzhou (%) R (S)	Zhangye (%) R (S)	Xining (%) R (S)	Yushu (%) R (S)	Ningxia (%) R (S)
FOX	33.3 (66.7)	0.00 (100)	37.5 (62.5)	80.0 (20.0)	0.00 (100)
ERY	100 (0.00)	85.7 (14.3)	75.0 (25.0)	100 (0.00)	100 (0.00)
TET	0.00 (100)	0.00 (100)	0.00 (100)	0.00 (100)	0.00 (100)
GEN	8.33 (91.67)	57.1 (42.9)	0.00 (100)	80.0 (20.0)	0.00 (100)
CIP	41. 6 (58.4)	57.1 (42.9)	12.5 (87.8)	80.0 (20.0)	42.9 (57.1)
VAN	0.00 (100)	0.00 (100)	0.00 (100)	0.00 (100)	0.00 (100)
SXT	50.0 (50.0)	42.8 (57.2)	87.5 (12.5)	20.0 (80.0)	42.9 (57.1)
FFC	8.33 (91.67)	14.3 (85.7)	25.0 (75.0)	80.0 (20.0)	57.1 (42.9)
LZD	0.00 (100)	0.00 (100)	0.00 (100)	0.00 (100)	0.00 (100)

R, Resistant; S, Susceptible; FOX, Cefoxitin; ERY, Erythromycin; TET, Tetracycline; GEN, Gentamicin; CIP, Ciprofloxacin; VAN, Vancomycin; SXT, Trimethoprim-Sulfamethoxazole; FFC, Florfenicol; and LZD, Linezolid.

### 3.3 Multi-drug resistant *S. haemolyticus* strains

**Figure 3:**
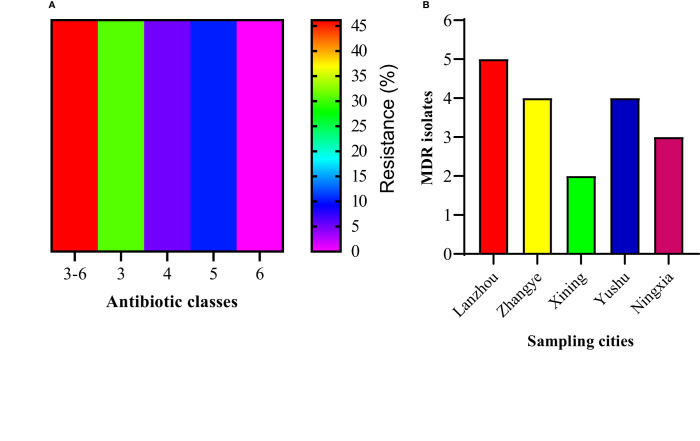
The percentage of MDR *S. haemolyticus.* **(A)** The percentage of *S. haemolyticus* strains showing resistance to three, four, five, six, and three to six classes of antibiotics. **(B)** The percentage distribution of MDR *S. haemolyticus* strains among different sampling sites.

The current study depicted 46.15% (18/39) MDR *S. haemolyticus* strains that showed resistance to at least one antimicrobial agent from ≥3 classes of antimicrobials. Among MDR strains, 30.77% (12/39) were resistant to three antibiotic classes, followed by 10.26% (4/39) resistant to five classes, and 5.13% (2/39) resistant to four classes. None of the strains (0.0%) showed resistance to all six classes of antibiotics (**Figure 3A**). The percentage of MDR strains was noted to be highest at 12.82% (5/39) from Lanzhou, followed by an equal percentage of 10.26% (4/39) from Zhangye and Yushu. The MDR strains were noted to be 7.69% (3/39) from Ningxia and 5.13% (2/39) from Xining (**Figure 3B**).

### 3.4 Genotypic AMR characteristics of *S. haemolyticus* strains

**Figure 4:**
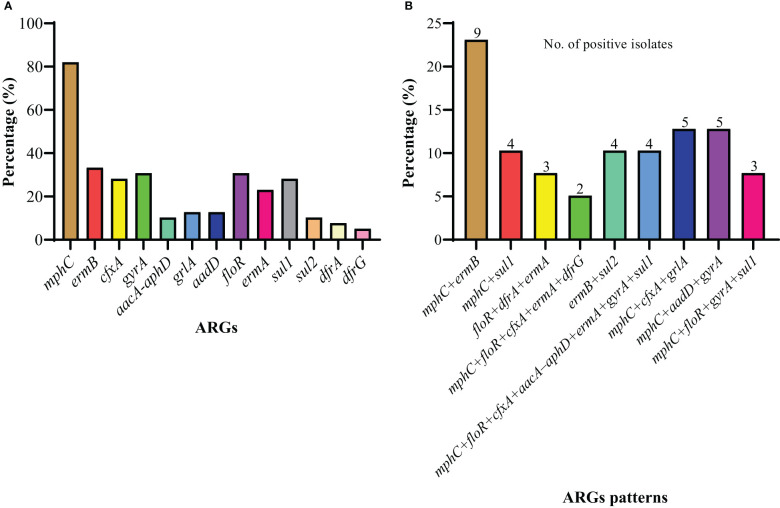
The distribution of ARGs among 39 *S. haemolyticus* strains. **(A)** The individual gene distribution **(B)** The different patterns of ARGs possessed by strains.

A total of 30 ARGs belonged to nine different antimicrobials and classes; *erm*A, *erm*B, *erm*C, *erm*F, *erm*(33), *mph*C, and *msr*B for erythromycin (macrolides, lincosamides, and streptogramin B); *van*A and *van*B for vancomycin (glycopeptide); *cfr* for linezolid (oxazolidinones); *cfx*A for cefoxitin (cephalosporin); *tet*M, *tet*O, *tet*L, and *tet*K for tetracycline (tetracycline’s); *fex*A and *flo*R for florfenicol (amphenicol); *aac*A-*aph*D and *aad*D for gentamicin (aminoglycosides); *gyr*A, *gyr*B, *grl*A, and *grl*B for ciprofloxacin (fluoroquinolones); and *sul1*, *sul2*, *sul3*, *dfr*A, *dfr*D, *dfr*G, and *dfr*K for trimethoprim-sulfamethoxazole (sulfonamides) were screened. Among 30 ARGs, 12 genes from different classes and multiple genes acquiring spectrum were identified. Among macrolides, lincosamides, and streptogramin B resistance genes, *mph*C was most predominant (82.05%), followed by *erm*B (33.33%) and *erm*A (23.08%), while *erm*C, *erm*F*, erm*(33), and *msr*B genes were not identified. Among amphenicol-resistance genes, only the *flo*R gene was harbored by 30.77% of strains. Among the aminoglycosides, the *aad*D gene was a little more prevalent (12.82%) than *aac*A-*aph*D (10.26%). The prevalence of the *gyr*A gene was noted to be higher (30.77%) compared to *grl*A (12.82%), with no identification of *gyr*B and *grl*B among the fluoroquinolones. However, none of the *S. haemolyticus* strains was harboring the glycopeptides, oxazolidinones, and tetracycline-resistance genes. Moreover, among sulfonamides, *sul1* (28.21%) was predominant, followed by *sul2* (10.26%), *dfr*A (7.69%), and *dfr*G (5.13%), while none of the strains was carrying the *sul3*, *dfr*D, and *dfr*K genes (**Figure 4A**). All of the *S. haemolyticus* strains were carrying the multiple genes spectrum, and most of the strains (43.6%, 17/39) were carrying the three different patterns of two ARGs: *mph*C+*erm*B (23.1%, 9/39), *mph*C+*sul1* (10.3%, 4/39), and *erm*B+*sul2* (10.3%, 4/39), followed by three different patterns of three ARGs; *flo*R+*dfr*A+*erm*A (7.7%, 3/39), *mph*C+*cfx*A+*grl*A (12.8%, 5/39), and *mphC*+*aadD*+*gyr*A (5/39). Moreover, 7.69% (3/39), 5.13% (2/39), and 10.3% (4/39) were carrying the single pattern of four, five, and seven ARGs (**Figure 4B**).

### 3.5 The comparison between phenotypic and genotypic resistance profile

The comparison in the phenotypic and genotypic expression of 39 *S. haemolyticus* strains is presented in **Table 3**. This comparative analysis was done with the perspective to analyze the expressions of *S. haemolyticus* strains and whether the strains showing resistance phenotypically were also carrying the ARGs or not and vice versa. The study noted unique phenotypic and genotypic expressions for all antibiotics except erythromycin. The number of phenotypically resistant strains was noted at 36/39 (92.31%) while the genotypic analysis showed that few strains were carrying the multiple-resistance genes. All of the strains showed no phenotypic and genotypic resistance to tetracycline, vancomycin, and linezolid.

**Table 3:** The comparison between phenotypic and genotypic resistance expressions.

Phenotype			Genotypes		
Antimicrobial agents	No. Positive (n= 39)	Percentage (%)	ARGs	No. Positive (n= 39)	Percentage (%)
Cefoxitin	11	28.21	*cfx*A	11	28.21
Tetracycline	0	0.00	*tet*M	0	0.00
			*tet*O	0	0.00
			*tet*L	0	0.00
			*tet*K	0	0.00
Erythromycin	36	92.31	*erm*A	9	23.08
			*erm*B	13	33.33
			*erm*C	0	0.00
			*erm*F	0	0.00
			*erm(33)*	0	0.00
			*mph*C	32	82.05
			*msr*A	0	0.00
Gentamicin	9	23.08	*aac*A*-aph*D	4	10.26
			*aad*D	5	12.82
Vancomycin	0	0	*van*A	0	0.00
			*van*B	0	0.00
Linezolid	0	0	*cfr*	0	0.00
Trimethorpim-Sulfamethoxazole	20	51.28	*sul1*	11	28.21
			*sul2*	4	10.26
			*sul3*	0	0.00
			*dfrA*	3	7.69
			*dfrD*	0	0.00
			*dfrK*	0	0.00
			*dfrG*	2	5.13
Ciprofloxacin	17	43.59	*gyr*A	12	30.77
			*gyr*B	0	0.00
			*grl*A	5	12.82
			*glr*B	0	0.00
Florfenicol	12	30.77	*flo*R	12	30.77
			*fex*A	0	0.00

### 3.6 The phylogenetic grouping of *S. haemolyticus* strains

**Figure 5:**
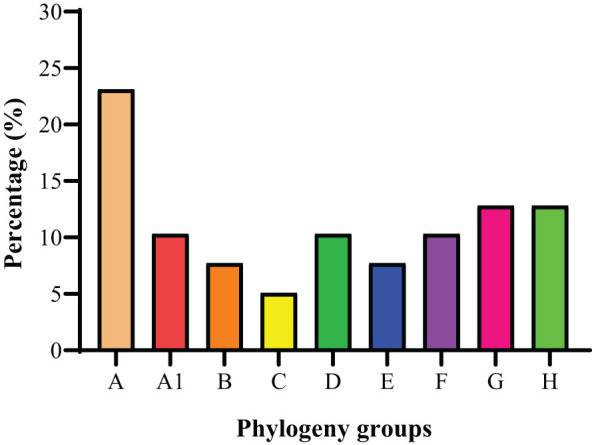
The phylogenetic grouping of 39 *S. haemolyticus* strains.

The phylogenetic grouping of 39 *S. haemolyticus* strains determined by PFGE generated by *Sma*I digestion and multiple PFGE patterns was observed among the strains. The BioNumerics cluster analysis software was used to construct the phylogenetic tree (**Figure S1**). The 39 *S. haemolyticus* were classified into eight different phylogenetic groups A, A1, B, C, D, E, F, G, and H. It was observed that most of the *S. haemolyticus* strains (23.1%, 9/39) belonged to group A and the least belonged (5.1%, 2/39) to group C. Moreover, 10.3% (4/39) were classified A1, D, and F, each, and 7.7% (3/39) into groups B and E, each. The percentage of strains categorized under groups G and H was noted to be 12.8% (5/39) in each group (**Figure 5**). In the phylogenetic grouping of strains among the different sampling cities, most of the strains from Lanzhou, Zhangye, Xining, Yushu, and Ningxia belonged to groups A, H, D, F, and E, respectively. Moreover, the phylogenetic grouping of *S. haemolyticus* strains from different cities was as follows: Lanzhou (A, C, G, and H), Zhnagye (A, B, and H), Xining (A, A1, B, D, and G), Yushu (A1 and F), and Ningxia (A1, C, D, and E) (**Table 4**).

**Table 4:** The phylogenetic grouping of 39 *S. haemolyticus* strains among different sampling cities.

Phylogenetic group	Lanzhou (n=12) No. (%)	Zhangye (n= 7) No. (%)	Xining (n= 8) No. (%)	Yushu (n= 5) No. (%)	Ningxia (n= 7) No. (%)
A	6 (50.0)	2 (28.6)	1 (12.5)	0 (0.0)	0 (0.0)
A1	0 (0.0)	0 (0.0)	1 (12.5)	1 (20.0)	2 (28.6)
B	0 (0.0)	1 (14.3)	2 (25.0)	0 (0.0)	0 (0.0)
C	1 (8.3)	0 (0.0)	0 (0.0)	0 (0.0)	1 (14.3)
D	0 (0.0)	0 (0.0)	3 (37.5)	0 (0.0)	1 (14.3)
E	0 (0.0)	0 (0.0)	0 (0.0)	0 (0.0)	3 (42.8)
F	0 (0.0)	0 (0.0)	0 (0.0)	4 (80.0)	0 (0.0)
G	4 (33.4)	0 (0.0)	1 (12.5)	0 (0.0)	0 (0.0)
H	1 (8.3)	4 (57.1)	0 (0.0)	0 (0.0)	0 (0.0)

### 3.7 Multi-locus sequence typing

**Figure 6:**
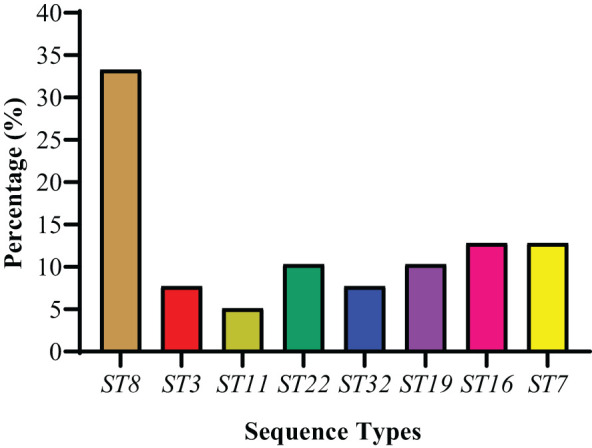
The percentage distribution of sequence types among 39 S. haemolyticus strains.

The MLST was done by the amplification and sequencing of seven housekeeping genes. Thirty-nine *S. haemolyticus* strains were categorized into eight different STs (ST8, ST3, ST11, ST22, ST32, ST19, ST16, and ST7). Among STs, ST8 was found to be dominant, accounting for 33.3% (13/39) (**Figure 6**). The percentage distribution of ST22 and ST19 were noted as similar (10.3%, 4/39 each). However, 5.1% (2/39) of strains were categorized as ST11, while the percentage of other STs, ST3, and ST32 were noted as 7.7% (3/39), followed by ST16 and ST7 as 12.8% (5/39) each (**Figure 6**). The detailed ST distribution in different sampling cities is given in **Table 5**. The dominant STs from Lanzhou, Zhangye, Xining, Yushu, and Ningxia were classified as ST8, ST7, ST22, ST19, and ST32, respectively. Moreover, the strains from Yushu only belonged to ST8 and ST19. The distribution of other STs identified in other cities was as follows: Lanzhou (ST11, ST16, ST7), Zhnagye (ST8, ST3), Xining (ST8, ST3, ST16), and Ningxia (ST8, ST11, ST22) (**Table 5**).

**Table 5:** MLST of 39 *S. haemlolyticus* strains from different sampling cities.

Sequence types	Lanzhou (n=12) No. (%)	Zhangye (n=7) No. (%)	Xining (n=8) No. (%)	Yushu (n=5) No. (%)	Ningxia (n=7) No. (%)
ST8	6 (50.0)	2 (28.6)	2 (25.0)	1 (20.0)	2 (28.6)
ST3	0 (0.0)	1 (14.3)	2 (25.0)	0 (0.0)	0 (0.0)
ST11	1 (8.3)	0 (0.0)	0 (0.0)	0 (0.0)	1 (14.3)
ST22	0 (0.0)	0 (0.0)	3 (37.5)	0 (0.0)	1 (14.3)
ST32	0 (0.0)	0 (0.0)	0 (0.0)	0 (0.0)	3 (42.8)
ST19	0 (0.0)	0 (0.0)	0 (0.0)	4 (80.0)	0 (0.0)
ST16	4 (33.4)	0 (0.0)	1 (12.5)	0 (0.0)	0 (0.0)
ST7	1 (8.3)	4 (57.1)	0 (0.0)	0 (0.0)	0 (0.0)

### 3.8 The association between STs, phylogenetic groups, and ARGs patterns

Most of the *S. haemolyticus* strains belonged to ST8 (13/39), and the phylogenetic group A+A1 was carrying the *mph*C*+erm*B and *mph*C*+sul1* gene patterns. Moreover, 10.3% of strains belonged to phylogenetic groups D and F with ST22 and ST19 carrying the *ermB+sul2* and *mph*C*+flo*R*+cfx*A*+aac*A*–aph*D*+erm*A*+gyr*A+*sul1* gene patterns, respectively. The least number of strains, 2/39 (5.1%) classified under ST11 and group C, were carrying the *mph*C*+flo*R*+cfx*A*+erm*A+*dfr*G resistance gene pattern. However, an equal number of strains 5/39 (12.8%) that were classified into ST16 and ST7 belonging to phylogenetic groups G and H harbored the *mph*C*+cfx*A*+grl*A and *mph*C*+aad*D*+gyr*A gene patterns, respectively (**Table 6**).

**Table 6:** The association between STs, phylogenetic groups, and ARGs patterns.

Phylogenetic groups	Gene patterns	STs	No. Positive (%)
A	*mph*C*+erm*B	ST8	9/39 (23.1%)
A1	*mph*C*+sul1*	ST8	4/39 (10.3%)
B	*flo*R*+dfr*A*+erm*A	ST3	3/39 (7.7%)
C	*mph*C*+flo*R*+cfx*A*+erm*A+*dfr*G	ST11	2/39 (5.1%)
D	*ermB+sul2*	ST22	4/39 (10.3%)
E	*mphC+flo*R*+gyrA*+*sul1*	ST32	3/39 (7.7%)
F	*mph*C*+flo*R*+cfx*A*+aac*A*–aph*D*+erm*A*+gyr*A+*sul1*	ST19	4/39 (10.3%)
G	*mph*C*+cfx*A*+grl*A	ST16	5/39 (12.8%)
H	*mph*C*+aad*D*+gyr*A	ST7	5/39 (12.8%)

## 4 Discussion

As per our knowledge and literature search, the current study is the first study reporting the prevalence and characterization of *S. haemolyticus* strains isolated from dairy cattle milk in Northwest, China, with a large sample size (n= 356). In the present study, 180/356 (50.6%) isolates were recovered and identified as *Staphylococcus* species confirming them as the major pathogen of dairy mastitis in China (Gao et al., 2017; He et al., 2020; Song et al., 2020). The prevalence of *S. aureus* was noted to be 14.9% (53/356) and NAS was 35.7% (127/356). Among the NAS, the prevalence of *S. haemolyticus* was noted to be 11.0% (39/356), while other NAS species were as follows: *S. sciuri* 10.4% (37/356), *S. saprophyticus* 7.6% (27/356), *S. chromogenes* 4.2% (15/356), *S. simulans* 1.4% (5/356), and *S. epidermidis* 1.1% (4/356) based on the bacteriological examination by the VITEK-2 system, as done previously (Gizaw et al., 2020; Chajęcka-Wierzchowska et al., 2023). Further confirmation of the targeted specie of *S. haemolyticus* strains was done by 16S rRNA gene sequencing, which is known to be a reliable molecular technique for microbial identification (Johnson et al., 2019; Han et al., 2020). The present study identified 39/356 (11.0%) strains as *S. haemolyticus*, which is consistent with previous findings (Klimiene et al., 2016). The current study identified *S. haemolyticus*, *S. sciuri*, *S. saprophyticus*, *S. chromogenes*, *S. simulans*, and *S. epidermidis* as the most frequently isolated NAS species from mastitic milk of dairy cows, which is consistent with previous studies from other regions of China (Qu et al., 2019) and other countries (Vanderhaeghen et al., 2014; Goetz et al., 2017; Jenkins et al., 2019). Moreover, in this study, the prevalence of *S. aureus* was noted to be 14.9% (53/356), which is higher than other *Staphylococcus* species. These results are consistent with a previously reported study that *S. aureus* is the major pathogen among *Staphylococcus* species associated with cow mastitis (Cortimiglia et al., 2016). However, many previous studies have reported the prevalence and characterization of *S. aureus* isolated from cow milk from different provinces in Northwest, China (Li et al., 2015; Li and Zhao, 2018; Dan et al., 2019; Kou et al., 2021; Shi et al., 2021). Our study only focused on the epidemiology of NAS, while other factors such as lactation stage, milking practices, and severity of disease-related factors such as somatic cell count were not recorded. The previous studies hypothesized that prior mentioned factors along with immunity level, nutrition, and farm management practices may be the predisposing factors for opportunistic pathogens such as NAS to invade and proliferate within the udder, leading to inflammation with clinical signs (Pal et al., 2019; Qu et al., 2019; Libera et al., 2021). Except for these factors, NAS species are also found to be ubiquitous in environmental settings such as soil, dust, and farm environment which may be the possible cause of udder infection by environmental opportunistic pathogens such as NAS (Schoenfelder et al., 2017). Adkins et al. (2018) previously documented that the intra-mammary (IM) infection of NAS species leads to subclinical mastitis with an increase in somatic cell count and later to clinical mastitis resulting in reduced milk yield. The control of NAS is essential and complicated because too many species are responsible for this problem. However, the implementation of proper mastitis prevention and control measures is guided by the National Mastitis Council (2017) to control the IM infections caused by NAS in dairy cows.

In this study, we analyzed the susceptibility of 39 *S. haemolyticus* strains of dairy origin against the nine antimicrobial agents, which are cefoxitin, tetracycline, vancomycin, linezolid, erythromycin, gentamicin, ciprofloxacin, trimethoprim-sulfamethoxazole, and florfenicol. Overall, most of the strains were found to be resistant to erythromycin (92.3%), followed by trimethoprim-sulfamethoxazole (51.3%), ciprofloxacin (43.6%), florfenicol (30.8%), cefoxitin (28.2%), and gentamicin (23.1%). The increased resistance to these antibiotics by *Staphylococcus* species has also been reported previously because of the increased use of these antibiotics in the treatment of NAS infections (Botrel et al., 2010; Persson et al., 2011; Qian et al., 2015; Kim et al., 2019). A similar study conducted by Gizaw et al. (2020) using archived isolates from cattle, farm environment, and personals noted a 3.6% resistance to ciprofloxacin, 12.5% to kanamycin, and 55.4% to erythromycin, which is much lower than the current findings, indicating the extensive use of these antibiotics at the sampled dairy farms in Northwest, China. Moreover, in the current study, the cefoxitin resistance was noted to be lower compared to the previous findings (Gizaw et al., 2020). The variations in antimicrobial resistance in different studies may be due to the highly genetically heterogeneous nature of bacterial isolates, which may exhibit varying degrees of phenotypic and genotypic expressions (Pacha et al., 2021). Moreover, another reason for the difference in susceptibilities might be the difference in susceptibility assay, *Staphylococcus* specie, and isolation source because according to our knowledge, none of the studies specifically check the antimicrobial response of *S. haemolyticus* strains isolated from dairy milk in China. Furthermore, all of the 39 *S. haemolyticus* strains were 100% susceptible to tetracycline, vancomycin, and linezolid, which is consistent with previous studies conducted by Kim et al. (2019) and Mello et al. (2020). Moreover, in this study, 46.15% of strains were MDR *S. haemolyticus* with 30.77% resistant to three antimicrobial classes, followed by 10.26% and 5.13% resistance to four and five classes, respectively. The irregular and non-justified use of antibiotics in livestock, especially in dairy production systems leads to the emergence of bacterial strains resistant to multiple antibiotics, which poses a potential threat to animal and human health (Phophi et al., 2019; Attallah et al., 2021). This misuse of antimicrobials put unnatural selective pressure on bacteria, which accelerates the evolution of resistant strains (Fair and Tor, 2014). Therefore, the justified use of antimicrobials in dairy production must be ensured and monitored through surveillance studies to get updated information regarding antimicrobial resistance. Moreover, the antimicrobial resistance can be lowered by restricting the use of antimicrobials as a feed supplement in production systems and finding alternative sources such as herbal medicine, essential oils, and traditional Chinese veterinary medicine (Lu et al., 2008; Yang et al., 2019; Cheng and Han, 2020; Ajose et al., 2022).

Previously, it was documented that food animals, especially cattle, were recognized as the reservoir of drug-resistant bacteria carrying ARGs, which can be transmitted to humans *via* the food web (Bennani et al., 2020; Sivagami et al., 2020). To understand the mechanism of gene transfer and the emergence of multi-drug resistant strains within and across the bacterial species, the knowledge of ARGs carried by pathogens is essential (Tóth et al., 2020). Therefore, the present study investigated the ARGs carried by *S. haemolyticus* strains isolated from dairy cattle milk; 30 ARGs belonging to nine antimicrobial classes were screened. Among the 30 ARGs, 12 resistance genes were identified among 39 *S. haemolyticus* strains. Among the macrolides, lincosamides, and streptogramin B resistance genes, *mph*C was most predominant, followed by *erm*B and *erm*A, while *erm*C, *erm*F*, erm*(33), and *msr*B genes were not identified. The *erm* family of gene codes for methylases confers resistance to streptogramin B (Petinaki and Papagiannitsis, 2019), macrolides (Feßler et al., 2018b), and lincosamides (Feßler et al., 2018a), while the *mphC* gene code for phosphotransferase confers resistance only to macrolides (Chajęcka-Wierzchowska et al., 2023). Other macrolide genes such as *msr*A and B gene codes for transporter proteins that confer resistance to both streptogramin B and macrolides (Quezada-Aguiluz et al., 2022) were not identified in this study. A similar study done by Qu et al. (2019) also detected that the abovementioned genes carried by *Staphylococcus* species conferred resistance to macrolides, lincosamides, and streptogramin B with an additional *lnu*A gene. Among the cephalosporins, the *cfx*A gene was identified, which is also in line with the findings of Qu et al. (2019) and other previous studies (El-Ashker et al., 2020; Jose et al., 2020). The higher frequency of these genes from dairy isolates indicates the potential use of cephalosporins for the treatment of clinical mastitis on dairy farms in China. Previously, Qian et al. (2015) reported that β-lactam antimicrobial agents such as penicillin, cephalosporin, and monobactams were extensively used for the treatment of bovine mastitis caused by *Staphylococcus* species. Among the amphenicol ARGs, only *the flo*R gene was harbored by *S. haemolyticus* strains, which is in accordance with the previously published study by Wu et al. (2021). Florfenicol is one of the most commonly used antimicrobials in the dairy sector because of its wide range of antimicrobial activity (Kawalek et al., 2016). Florfenicol acts by binding with the 50S ribosomal subunit, resulting in the inhibition of protein synthesis. *floR* gene encodes for membrane-associated proteins that confer resistance to florfenicol (Shore et al., 2016). Another study conducted by Li et al. (2020) confirmed that *flo*R was found to be the predominant gene conferring resistance to florfenicol in different animal-derived bacteria. Among the aminoglycosides, both *aad*D and *aac*A-*aph*D genes were identified, which is in line with previously published studies (Qu et al., 2019; Kowalewicz et al., 2023). The *aac*A-*aph*D gene code for a multifunctional enzyme confers resistance to multiple aminoglycosides such as amikacin, gentamicin, tobramycin, and kanamycin (Schwarz et al., 2018), while *aad*D gene codes for adenyltransferase enzyme confer resistance to neomycin, kanamycin, tobramycin, and paromomycin (Batool et al., 2021). The prevalence of the *gyr*A gene was noted to be higher compared to *grl*A among the fluoroquinolones that confer resistance to ciprofloxacin, as studied previously (Bonsaglia et al., 2018; Pham et al., 2019). The acquisition of *gyr*A/B and *grl*A/B genes by *Staphylococcus* species confers resistance to fluoroquinolones because they code for two enzymes, DNA gyrase and topoisomerase IV, respectively, which are required by the bacteria during DNA replication (Schwarz et al., 2018; Lapointe et al., 2021). However, none of the *S. haemolyticus* strains was harboring the glycopeptides, oxazolidinones, and tetracycline-resistance genes, which is contrary to the findings of Qu et al. (2019) but inconsistent with the findings of Liu et al. (2022) for tetracycline. Among sulfonamides, *sul1* was predominant, followed by *sul2*, *dfr*A, and *dfr*G, while none of the strains was carrying the *sul3*, *dfr*D, and *dfr*K genes. A study conducted by Nurjadi et al. (2021) also identified that *dfr*A, *dfr*D, *dfr*G, and *dfr*K genes confer resistance to trimethoprim, and Khan et al. (2022) detected *sul1*, *sul2*, and *sul3* genes that confer resistance to sulfonamides among the *Staphylococcus* species. The *dfr* genes confer resistance to trimethoprim by modifications in the target dihydrofolate reductase enzyme (Nurjadi et al., 2021). All of the *S. haemolyticus* strains were carrying the multiple gene patterns that are mentioned in **Figure 5B**, which follows many gene patterns identified in a previous study (Qu et al., 2019). This could be explained by the fact that a single resistance phenotype can be mediated by multiple resistance genes (Varela et al., 2021). The presence of diverse ARGs in mastitic milk of dairy cows poses a potential threat to the transmission of these genes from animal to human microbiota through horizontal gene transfer (HGT) by mobile genetic elements (Argudín et al., 2017; Hu et al., 2017). Therefore, it is necessary to monitor antimicrobial resistance, and their transmission mechanisms are of much significance to guide the rational use of antimicrobials in dairy production.

The PFGE and MLST methods are still used for the genotypic characterization of bacterial isolates and to track the dissemination of infections with limitations (Neoh et al., 2019; He and Reed, 2020; Pizauro et al., 2021). Therefore, the present study used these methods for the genotypic characterization of 39 *S. haemolyticus* strains and grouped strains into eight phylogenetic groups and sequence types. Based on PFGE, most of the strains belonged to group A (9/39), followed by G (5/39), H (5/39), A1 (4/39), D (4/39), F (4/39), B (3/39), E (3/39), and C (2/39). Most of the strains belonging to group A are consistent with the previous findings of Qu et al. (2019). Previously, a study conducted by Naushad et al. (2016) also grouped NAS species into five: A, B, C, D, and E phylogenetic groups based on whole genome sequencing (WGS). It was observed that phylogenetic groups were sharing a common cluster among 39 *S. haemolyticus* strains isolated from the close locality. The study noted that the farms in a close geographical area may share the same genotypes, suggesting clonal transmission between dairy farms (Rainard et al., 2018). Notably, the MLST results were found to be consistent with the results of PFGE as each ST type corresponded to a single PFGE type. Among the STs, eight unrelated STs were identified with ST8 being predominant, followed by ST7, ST16, ST22, ST19, ST3, ST32, and ST11. Previously, multiple studies from human and animal settings reported ST8 as an emerging sporadic clone among *Staphylococcus* species from different regions of mainland China (Li et al., 2018; Liu et al., 2018; Dong et al., 2020). The other identified STs in this study have also been reported previously among the *Staphylococcus* species from China: ST7 (Ou et al., 2020; Zhu et al., 2022; Gu et al., 2023), ST 3 (Chang et al., 2022; Lin et al., 2022), ST 16 (Du et al., 2013), ST22 (Zhou et al., 2022; Gu et al., 2023), and ST19 (Jiang et al., 2016). As per our literature search, ST11 and ST32 were not reported previously among the *Staphylococcus* genus from China, while ST32 was reported in *the S. epidermidis* strain from the USA (Mendes et al., 2012). The presence of common STs in both animal and human strains is indicative of in-between species transfer of genetic heritability from both perspectives. The capability of a few pathogens from one host specie to another specie poses a potential hazard to human health and the food chain (Richardson et al., 2018). ST11 is reported in China in gram-negative bacteria, e.g., *K. pneumonia* and *S. enteritidis* strains (Li et al., 2021; Zhang et al., 2021). The identification of ST11 among *S. haemolyticus* strains in our study may indicate the possible transfer of genes from one bacterial specie to another specie in different ecological niches through horizontal gene transfer mechanisms such as transduction, conjugation, and transformation (Bolotin and Hershberg, 2017; Richardson et al., 2018). There is a need to conduct in-depth studies to understand more about virulence determinants, plasmid typing, serotyping, and gene transfer mechanisms within one health framework to understand the genetic basis of antimicrobial resistance spread and pathogenicity mechanisms using advanced characterization techniques such as whole genome sequencing (WGS).

## 5 Conclusions

The study noted a relatively high prevalence of *S. haemolyticus* and other *Staphylococcus* species at sampled dairy farms in Northwest, China, highlighting the importance of emerging antimicrobial-resistant pathogens affecting dairy herds. The antimicrobial susceptibility showed higher resistance to erythromycin, trimethoprim-sulfamethoxazole, gentamicin, cefoxitin, ciprofloxacin, and florfenicol, suggesting that the use of these antimicrobials at dairy farms needs to be closely monitored. However, susceptibility to tetracycline, vancomycin, and linezolid could be the antimicrobials used to treat mastitis caused by this pathogen. Most of the strains carrying the multiple resistance genes pose a potential threat to public health *via* the consumption of contaminated milk. The phylogenetic analysis classified strains from groups A-H, while MLST detected eight STs, with ST8 being the most predominant. These findings provided new insights into our understanding of the epidemiology and genetic characteristics of *S. haemolyticus* in dairy farms to inform interventions limiting the spread of AMR in dairy production.

## Data availability statement

The original contributions presented in the study are included in the article/**Supplementary Material**. Further inquiries can be directed to the corresponding author.

## Ethics statement

Ethical review and approval was not required for the animal study because the study did not involve any invasive procedure on animals. Written informed consent was obtained from the owners for the participation of their animals in this study.

## Author contributions

MS: Writing-original draft; JX and XM: Investigation and Methodology; ZW and XH: Data curation and Formal analysis; ZH, RS, and HZ: Writing-review and editing; WP: Conceptualization, Investigation, Funding acquisition, and Supervision. All authors contributed to the article and approved the submitted version.
