# Supplementary material for: Molecular epidemiology and characterization of antimicrobial-resistant Staphylococcus haemolyticus strains isolated from dairy cattle milk in Northwest, China
Source: Front Cell Infect Microbiol. 2023 May 17;13:1183390. doi: 10.3389/fcimb.2023.1183390 (PMC10230075; doi:10.3389/fcimb.2023.1183390)
Supplement: Supplementary file 1 [file DataSheet_1.docx]

**Table 1.** 16S rRNA gene primers information

| **Gene** | **Primer name** | **Primer sequences (5′-3′)** | **Amplicon size** | **Annealing temperature (℃)** | **Reference** |
| --- | --- | --- | --- | --- | --- |
| 16S rRNA | 16S-Forward | TCCAGATTACAACTTCACCAGG | 1500 | 55 | (Marincola et al., 2021) |
|  | 16S-Reverse | GTTCTGCAGTACCGGATTTGC |  |  |  |

**Table 2.** Antibiotic resistance gene primers and annealing temperatures

| **Antibiotic**  **Class** | **Antibiotic Name** | **Genes** | **Primer Sequences (5’----3’)** | **Annealing temperature (℃)** | **Amplicon size (bp)** | **References** |
| --- | --- | --- | --- | --- | --- | --- |
| Macrolides, Lincosamides, and Streptrogramin B | Erythromycin | *erm*A | **F:** GTTCAAGAACAATCAATACAGAG | 52 | 421 | (Qu et al., 2019) |
|  |  |  | **R:** GGATCAGGAAAAGGACATTTTAC |  |  |  |
|  |  | *erm*B | **F:** CCGTTTACGAAATTGGAACAGGTAAAGGGC | 55 | 359 |  |
|  |  |  | **R:** GAATCGAGAC TTGAGTGTGC |  |  |  |
|  |  | *erm*C | **F:** GCTAATATTGTTTAAATCGTCAATTCC | 52 | 572 |  |
|  |  |  | **R:** GGATCAGGAAAAGGACATTTTAC |  |  |  |
|  |  | *erm*F | **F:** TGGCATTACTTCCGATAT | 55 | 460 |  |
|  |  |  | **R:** GACAACTTCCAGCATTTC |  |  |  |
|  |  | *erm(33)* | **F:** TTGAAATTGGCTCAGGAA | 54 | 404 |  |
|  |  |  | **R:** TACACTTGGCTTAGGATG |  |  |  |
|  |  | *mph*C | **F:** TGGGAAATTGAACACAAACC | 52 | 510 |  |
|  |  |  | **R:** AGCAAACTCAGCGATAAACA |  |  |  |
|  |  | *msr*B | **F:** GGCACAATAAGAGTGTTTAAAGG | 50 | 940 |  |
|  |  |  | **R:** AAGTTATATCATGAATAGATTGTCCTGTT |  |  |  |
| Glycopeptide | Vancomycin | *van*A | **F:** AATACTGTTTGGGGGTTGCTC | 50 | 734 | (Westberg et al., 2022) |
|  |  |  | **R:** CTTTTTCCGGCTCGACTTCCT |  |  |  |
|  |  | *van*B | **F:** GCGGGGAGGATGGTGGGATAGAG | 50 | 420 |  |
|  |  |  | **R:** GGAAGATACCGTGGCTCAAAC |  |  |  |
| Oxazolidinones | Linezolid | *cfr* | **F:** TGAAGTATAAAGCAGGTTGGGAGTCA | 54 | 340 | (Gostev et al., 2021) |
|  |  |  | **R:** ACCATATAATTGACCACAAGCAGC |  |  |  |
| Cephalosporin | Cefoxitin | *cfx*A | **F:** CTGTAATATCGGAGGGTTTATTTTG | 50 | 938 | (Qu et al., 2019) |
|  |  |  | **R:** CAGTATTTATTATGCATTTAGAATA |  |  |  |
| Tetracycline’s | Tetracycline | *tet*M | **F:** GAACTCGAACAAGAGGAAAGC | 55 | 740 | (Qu et al., 2019) |
|  |  |  | **R:** ATGGAAGCCCAGAAAGGAT |  |  |  |
|  |  | *tet*O | **F:** AACTTAGGCATTCTGGCTCAC | 52 | 519 |  |
|  |  |  | **R:** TCCCACTGTTCCATATCGTCA |  |  |  |
|  |  | *tet*L | **F:** TGAACGTCTCATTACCTG | 50 | 993 |  |
|  |  |  | **R:** ACGAAAGCCCACCTAAAA |  |  |  |
|  |  | *tet*K | **F:** TCCTGGAACCATGAGTGT | 50 | 189 |  |
|  |  |  | **R:** AGATAATCCGCCCATAAC |  |  |  |
| Amphenicol | Florfenicol | *fex*A | **F:** TTGGGAAGAATGGTTCAGGG | 55 | 977 | (Wu et al., 2021) |
|  |  |  | **R:** ATCGGCTCAGTAGCATCACG |  |  |  |
|  |  | *floR* | **F*:*** ATGACCACCACACGCCCCGCGTGGGC | 58 | 1198 |  |
|  |  |  | **R*:*** CTTCGATCCCGCGACGTTCCTTCCGAGA |  |  |  |
| Aminoglycosides | Gentamicin | *aac*A*/aph*D | **F:** CAATAAGGGCATACCAAA | 56 | 211 | (Qu et al., 2019) |
|  |  |  | **R:** CTATCATAACCACTACCG |  |  |  |
|  |  | *aad*D | **F:** CTATTGGTGTTTATGGCCT | 54 | 630 |  |
|  |  |  | **R:** ATCCGTGTCGTTCTGTCC |  |  |  |
| Fluoroquinolones | Ciprofloxacin | *gyr*A | **F:** TGGCTGAATTACCTCAATCAAG | 51 | 750 | (Bonsaglia et al., 2018) |
|  |  |  | **R:** CTTCAATAACTGCACGAGAACG |  |  |  |
|  |  | *gyr*B | **F:** ATACACGTGAAGGTATGACAGC | 47 | 779 |  |
|  |  |  | **R:** TCCAAGACCTTTGTATCGTGCA |  |  |  |
|  |  | *grl*A | **F:** TTAGGTGATCGCTTTGGAAGAT | 62 | 472 |  |
|  |  |  | **R:** TACCTGTAGAACCATTCACTAG |  |  |  |
|  |  | *grl*B | **F:** ACTTCTGAAGCTAGAAGTGCTG | 56 | 754 |  |
|  |  |  | **R:** TTTCTGGGTTCATCGTCGTTTC |  |  |  |
| Sulfonamides | Sulfamethoxazole/Trimethoprim | *dfr*A | **F-**CACTTGTAATGGCACGGAAA | 57 | 270 | (Manoharan et al., 2021) |
|  |  |  | **R-**CGAATGTGTATGGTGGAAAG |  |  |  |
|  |  | *dfr*D | **F-**CCCTGCTATTAAAGCACC | 57 | 340 |  |
|  |  |  | **R-**CATGACCAGATAACTC |  |  |  |
|  |  | *dfr*G | **F-**TGCTGCGATGGATAAGAA | 57 | 405 |  |
|  |  |  | **R-**TGGGCAAATACCTCATTCC |  |  |  |
|  |  | *dfr*K | **F-**CAAGAGATAAGGGGTTCAGC | 57 | 229 |  |
|  |  |  | **R-**ACAGATACTTCGTTCCACTC |  |  |  |


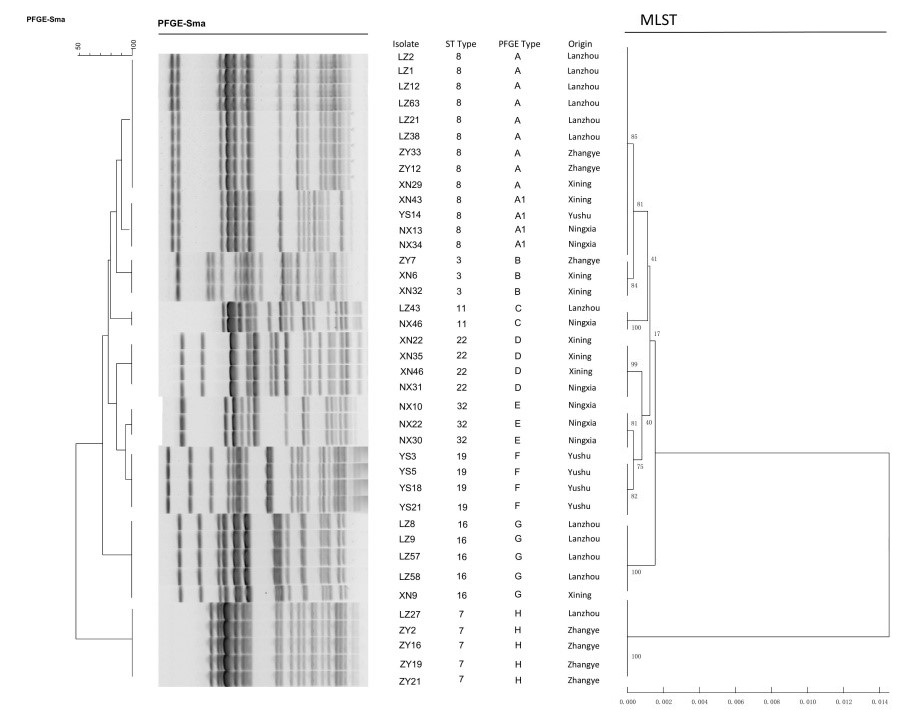


**Figure 1.** PFGE patterns and MLST types of *S. haemolyticus* isolates. UPGMA phylogenetic tree of the concatenated MLST sequences of 39 Strains of *Staphylococcus haemolytic*, the confidence level of the evolutionary tree, the higher the credibility is higher, who get closer to the higher homology

**References**

Bonsaglia, E., Silva, N., Rossi, B., Camargo, C., Dantas, S., Langoni, H., et al. (2018). Molecular epidemiology of methicillin-susceptible Staphylococcus aureus (MSSA) isolated from milk of cows with subclinical mastitis. *Microbial pathogenesis* 124**,** 130-135.

Gostev, V., Leyn, S., Kruglov, A., Likholetova, D., Kalinogorskaya, O., Baykina, M., et al. (2021). Global expansion of linezolid-resistant coagulase-negative staphylococci. *Frontiers in Microbiology* 12**,** 661798.

Manoharan, M., Sistla, S., and Ray, P. (2021). Prevalence and molecular determinants of antimicrobial resistance in clinical isolates of Staphylococcus haemolyticus from India. *Microbial Drug Resistance* 27(4)**,** 501-508.

Marincola, G., Liong, O., Schoen, C., Abouelfetouh, A., Hamdy, A., Wencker, F.D., et al. (2021). Antimicrobial resistance profiles of coagulase-negative staphylococci in community-based healthy individuals in Germany. *Frontiers in Public Health* 9**,** 684456.

Qu, Y., Zhao, H., Nobrega, D.B., Cobo, E.R., Han, B., Zhao, Z., et al. (2019). Molecular epidemiology and distribution of antimicrobial resistance genes of Staphylococcus species isolated from Chinese dairy cows with clinical mastitis. *Journal of dairy science* 102(2)**,** 1571-1583.

Westberg, R., Stegger, M., and Söderquist, B. (2022). Molecular Epidemiology of Neonatal-Associated Staphylococcus haemolyticus Reveals Endemic Outbreak. *Microbiology spectrum* 10(6)**,** e02452-02422.

Wu, C., Zhang, X., Liang, J., Li, Q., Lin, H., Lin, C., et al. (2021). Characterization of florfenicol resistance genes in the coagulase-negative Staphylococcus (CoNS) isolates and genomic features of a multidrug-resistant Staphylococcus lentus strain H29. *Antimicrobial Resistance & Infection Control* 10(1)**,** 1-10.
